# Diseases of Women

**Published:** 1849-01

**Authors:** 


*Diseases of Women.—Displacements of the Uterus.*—Dr. Bell, of Glasgow, writes a very interesting article on this subject in the Monthly Journal of Medical Science.—First:—As to *Retroversion* a condition, which he states to be of frequent occurrence; that parturition is a frequent cause, except in those cases which have not been pregnant. In these latter cases, enlarged uterus, or long-standing dysmenorrhœa, *particularly dysmenorrhœa*, always tends to produce enlargement, and thereby leads to abnormal positions. *Anteversion*, though esteemed by French writers as the most common affection of uterine displacements, yet Dr. Bell has seen but few cases. *Retroflexion*, doubted by many writers, Dr. Bell considers it of frequent occurrence. The causes of this and anteversion are pretty nearly the same as retroversion. Denman's account of the latter displacement is very correct. *Anteflexion* stands in the same relation to *antiversion* as *retroflexion* to *retroversion*. Treatment most judicious must be to restore the organ to its normal size and position. The mere restoring the position is not sufficient, for it will soon relapse, if the condition be not removed. Remove the congestion and hypertrophy, and the organ will resume its position. It is often impossible to restore its position, even temporarily, from its size. Dr. Bell, therefore, prefers removing congestion and inflammatory action before replacement. Judging from his own practice, Dr. Bell considers few cases require mechanical assistance. The principal objects to be attended to in treatment are, recumbent position, regular state of the bowels, local depletion, mercurials, and lastly, mechanical treatment, by supports, pessaries, &c. Dr. Bell does not appear sanguine of the mechanical means, and has succeeded in effecting cures by medicine where mechanical means have failed. This valuable paper concludes with some interesting cases in favor of medical treatment.

